# Enhanced Transdermal Immunization via Solid-in-Oil Nanodispersions Incorporating Dendritic Cell-Targeting Peptide

**DOI:** 10.3390/molecules31152668

**Published:** 2026-07-31

**Authors:** Md Samiul Islam, Md. Shahin Sarker, Yoshirou Kawaguchi, Rie Wakabayashi, Noriho Kamiya, Muhammad Moniruzzaman, Masahiro Goto

**Affiliations:** 1 Department of Applied Chemistry, Graduate School of Engineering, Kyushu University, 744 Motooka, Fukuoka 819-0395, Japan; islam.md.samiul.333@s.kyushu-u.ac.jp (M.S.I.); sarker.md.shahin.772@s.kyushu-u.ac.jp (M.S.S.); kawaguchi.yoshirou.168@m.kyushu-u.ac.jp (Y.K.); wakabayashi.rie.122@m.kyushu-u.ac.jp (R.W.); kamiya.noriho.367@m.kyushu-u.ac.jp (N.K.); 2 Department of Pharmaceutical Technology, Faculty of Pharmacy, University of Dhaka, Dhaka 1000, Bangladesh; 3 Department of Pharmacy, Jashore University of Science and Technology, Jashore 7408, Bangladesh; 4 Center for Future Chemistry, Kyushu University, 744 Motooka, Fukuoka 819-0395, Japan; 5 Mechanical Engineering Technology, CUNY New York City College of Technology, 186 Jay Street, Brooklyn, NY 11201, USA; muhammad.moniruzzaman43@citytech.cuny.edu

**Keywords:** transdermal immunization, solid-in-oil (S/O) nanodispersion, dendritic cells, ovalbumin, HR8 peptide

## Abstract

Transdermal immunization represents a promising needle-free alternative to conventional vaccination. However, efficient antigen delivery and robust immune activation remain major challenges. In this study, a solid-in-oil (S/O) nanodispersion system comprising a dendritic cell-targeting peptide (HR8), ovalbumin (OVA), and an adjuvant was developed for transdermal immunization. The HR8 peptide along with OVA was successfully incorporated into an S/O nanodispersion with an optimal hydrodynamic diameter of particles (<200 nm) and exhibited stable physical properties for up to 90 days. In vitro and in vivo studies demonstrated enhanced antigen delivery with insignificant skin irritation in C57BL/6N mice. Moreover, in vivo transdermal immunization studies demonstrated that the addition of the HR8 peptide enhanced OVA-specific IgG responses (~1.5-fold). Notably, the HR8 peptide also promoted an approximately 2.5-fold increase in IgG2c levels, suggesting a shift toward a more T helper type 1-biased immune response, as reflected by an increased IgG2c/IgG1 ratio. Overall, these findings demonstrate that the incorporation of HR8 peptide into an S/O nanodispersion enables efficient transdermal antigen delivery and improved immunogenicity, highlighting its potential as a simple, non-invasive, and patient-friendly immunization strategy.

## 1. Introduction

Immunization is a powerful strategy designed to elicit specific and durable immune responses to treat or cure diseases through the administration of specific antigens, including inactivated or attenuated pathogens, peptides, or proteins [1]. In the context of cancer therapy, vaccines that engage the adaptive immune system offer distinct advantages over conventional cancer therapies, such as sustained therapeutic efficacy and reduced systemic toxicity [2]. However, most vaccines are administered via injection, which introduces several limitations, including pain, the risk of needle-related injuries, and the need for trained healthcare professionals for proper administration [3]. These challenges have driven increasing interest in the development of safe, simple, and non-invasive delivery approaches via the oral, nasal [4,5] and transdermal routes [6,7,8].

Transdermal immunization has emerged as a potential approach because of its capacity to exploit the immunologically rich environment of the skin. This strategy utilizes the immune networks in the epidermis and dermis, where antigen-presenting cells (APCs) are more densely distributed than in the muscle or subcutaneous tissue, which allows the induction of strong immune responses at lower antigen doses [9]. In addition, transdermal delivery enables the convenient administration of antigens using patch-based systems. Among APCs, dendritic cells (DCs) play a pivotal role and are critical mediators of antigen presentation and the initiation of adaptive immunity. The presence of multiple DC subsets in the skin highlights the potential of transdermal immunization as an effective delivery strategy. Administering antigens via this route can also stimulate dermal DCs [10]. DCs are highly efficient and professional APCs that process and present antigens to activate T cells [11,12,13,14]. Therefore, immunotherapeutic approaches mediated by DCs are essential for efficient antigen presentation and adaptive immune activation. However, the insufficient targeting of DCs has significantly compromised the efficacy of immunization [15]. DC-targeted immunization can boost CD8^+^ T cell-mediated immunity resulting in T helper (Th) type 1 immune responses [16,17]. Among T helper cell subsets, Th1 and Th2 cells play distinct roles in immunity, with Th1 cells mediating cellular immunity and Th2 cells supporting humoral responses. However, a balanced Th1/Th2 response is critical for optimal therapeutic outcomes [18,19].

DC-targeted antibodies have been extensively explored as a strategy to enhance immunization because of their ability to achieve the precise and efficient delivery of antigens to DCs [20,21]. Recently, targeting peptides have emerged as an attractive alternative to antibodies because of their low molecular weight, high permeability, reduced immunogenicity, lower production costs, and versatility for co-delivery with specific antigens [22,23]. Regarding vaccine applications, peptides have been utilized to facilitate MHC class I-dependent antigen presentation by delivering exogenous antigens into the cytosol of APCs [24,25,26,27,28]. Despite these advantages, their clinical application is limited because of difficulties in delivery, poor stability, and low bioavailability [29]. However, Zheng et al. reported a notable advancement by identifying and optimizing a novel DC-targeting peptide with the D-amino acid sequence HLHKHHLR (HR8), using a phage display technique, which enhanced ovalbumin (OVA) delivery following its subcutaneous administration [15]. Zheng et al. used CpG oligodeoxynucleotide (ODN) 1826 as an adjuvant to activate DC via Toll-like receptor 9 (TLR9) signaling, thereby overcoming immune tolerance [30,31,32]. Nevertheless, subcutaneous administration has some drawbacks, including needle-related complications and possible pathogen transmission resulting from the improper reuse of needles and syringes [6]. Furthermore, the short half-life of peptides following subcutaneous administration requires repeated dosing, leading to pain, psychological discomfort, and ultimately reduced patient compliance [33].

To address these limitations, the solid-in-oil (S/O) nanodispersion system has recently gained attention as a versatile platform for transdermal antigen delivery. Previous studies have demonstrated that the S/O nanodispersion system can effectively deliver OVA across the skin, either alone or in combination with peptides and adjuvants [18,34,35,36]. Considering these advantages, the current study used an S/O nanodispersion system to co-deliver OVA with a DC-targeting peptide via the transdermal route. To the best of our knowledge, this study represents the first evidence of transdermal immunization enabled by the co-delivery of a DC-targeting peptide with a model antigen (OVA) through an S/O nanodispersion system. The present study developed an S/O nanodispersion system incorporating the DC-targeting HR8 peptide and OVA as a model antigen. The physical properties of various nanodispersions were characterized, and their ability to induce enhanced immune responses following transdermal immunization was evaluated.

## 2. Results and Discussion

### 2.1. Characterization of the S/O Nanodispersions with HR8 Peptide

**Figure 1:**
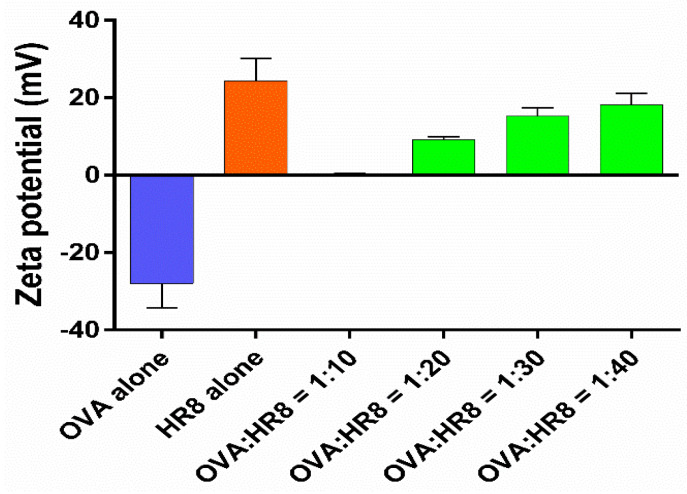
Zeta potentials of OVA alone, HR8 alone, and the OVA-HR8 complex at different molar ratios.

Prior to the preparation of the S/O nanodispersions, the complexation behavior between OVA and the HR8 peptide was investigated as previously reported with slight modifications [18]. The formation of stable antigen-peptide complexes is essential for their efficient incorporation into the S/O system and their transdermal delivery. To evaluate complex formation, aqueous solutions of OVA and HR8 peptide using Milli-Q water were prepared at molar ratios of 1:10, 1:20, 1:30, and 1:40 and their zeta potentials were measured using DLS (Figure 1). OVA alone had a negative surface charge (−28.0 ± 6.3 mV), whereas the HR8 peptide alone had a positive surface charge (24.4 ± 5.7) attributable to the presence of multiple histidine and lysine residues that confer cationic properties [15,37]. Upon complexation, the zeta potentials of the OVA-HR8 complexes shifted progressively from negative to positive values with an increase in HR8 content (Table S1). The gradual shift in zeta potential strongly indicates the formation of electrostatic interactions between negatively charged OVA and the positively charged HR8 peptide. Similar trends have been reported for protein-peptide or DNA-ionic liquid complexes where charge neutralization followed by charge reversal confirms successful complex formation [18,38]. Furthermore, the positive surface charge observed for these complexes suggests that OVA is effectively surrounded by HR8 peptides, which is advantageous for DC-targeting antigen delivery [38].

**Figure 2:**
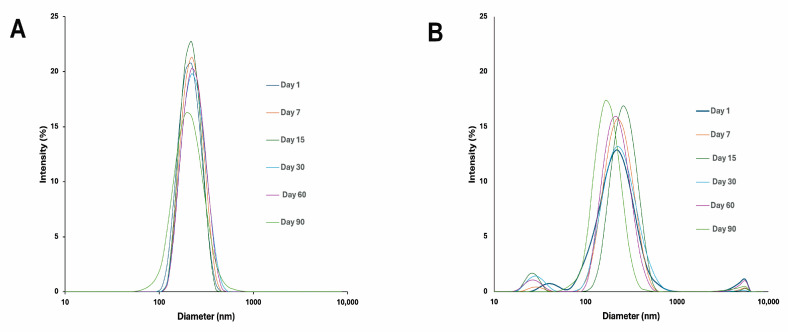
The hydrodynamic diameter of particles of F8-S/O nanodispersions (**A**) and F12 S/O nanodispersions (**B**) until 90 days.

The S/O nanodispersions were then prepared by mixing with a surfactant-containing cyclohexane solution at a ratio of 1:2 (*v*/*v*) followed by freeze-drying and the addition of 1 mL IPM. However, for optimization, two surfactants, L-195 and ER-290, were evaluated because of their ability to stabilize water in oil systems that facilitates transdermal delivery [34,39,40]. Twelve different S/O formulations (Tables S2 and S3) were prepared and characterized for the hydrodynamic diameter of particles and polydispersity index (PDI) using DLS. Among the tested formulations, F8-S/O (ER-290; 50 mg) and F12-S/O (ER-290; 100 mg) nanodispersions exhibited desirable physicochemical features, including nanoscale size (~200 nm) and low PDI values for about 90 days. The physical stability of the formulations was evaluated at room temperature, during which no significant changes in the hydrodynamic diameter of particles or PDI were observed for the F8-S/O and F12-S/O nanodispersions (Table S4, Figure 2). The maintenance of consistent hydrodynamic diameter of particles and low PDI values indicated that the S/O nanodispersions remained physically stable, with no evidence of aggregation, coalescence, or phase separation over the storage period. Such stability is critical for ensuring uniform antigen distribution, reproducible performance, and reliable transdermal delivery. Based on robust stability under ambient conditions, F8-S/O and F12-S/O were selected for subsequent in vitro and in vivo studies.

**Figure 3:**
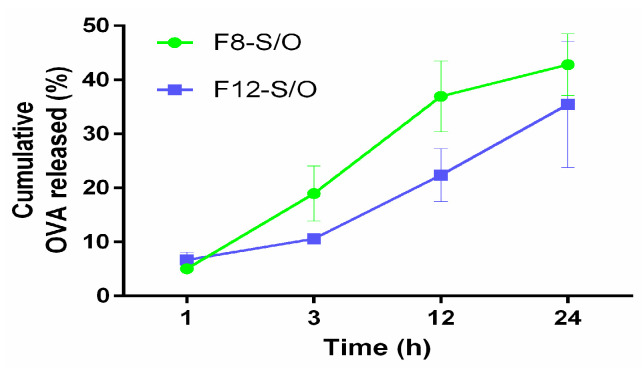
The cumulative release of OVA from S/O nanodispersions (F8-S/O and F12-S/O). Data presented as the mean ± SD (*n* = 3).

Next, the release of OVA from in vitro S/O nanodispersions was evaluated under hydrophilic conditions using phosphate-buffered saline (in PBS, 37 °C). The release profile was evaluated for 24 h to reflect the intended duration of transdermal patch application. As shown in Figure 3, F8-S/O and F12-S/O nanodispersions exhibited sustained and controlled release behavior throughout the 24 h period [41,42].

### 2.2. In Vitro Skin Permeability Evaluation

**Figure 4:**
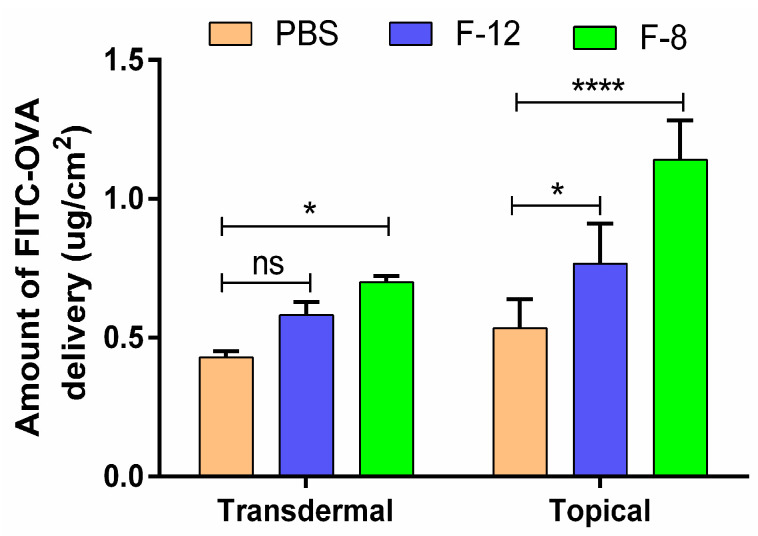
Amounts of FITC-OVA that permeated (transdermal) and penetrated (topical) through mouse skin. Statistical significance was assessed using Tukey’s multiple comparison test: * *p* < 0.05 and **** *p* < 0.0001. Values are the mean ± SD (*n* = 3). ns = non-significant.

In vitro skin permeation studies revealed that both S/O formulations markedly enhanced the delivery of FITC-OVA relative to the PBS control (Figure 4). It was reported that S/O nanodispersions permeated via the intercellular route, where IPM disrupts stratum corneum (SC) lipids, reducing barrier integrity and enhancing permeation [43,44]. In our study, the F8-S/O nanodispersion showed significantly increased permeation and penetration efficiency compared with that of the F12-S/O nanodispersion. These results may be attributed to the lower surfactant content in F8-S/O, which likely enables the more efficient release of antigen. In contrast, higher surfactant concentrations, as in F12-S/O, may result in stronger interactions between the antigen and surfactant molecules, thereby restricting the release of antigen. A study by Kitaoka et al. demonstrated that higher concentrations of surfactants limited antigen exposure by forming antigen–surfactant complexes, ultimately reducing the permeation and immunological efficacy [40].

### 2.3. In Vitro Biocompatibility Study

**Figure 5:**
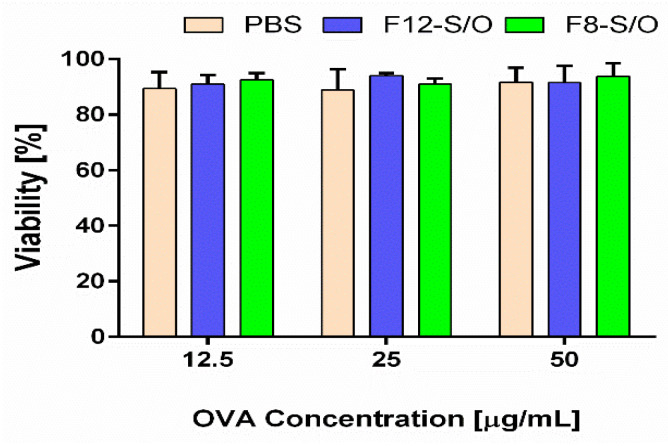
Cell viability was evaluated with different formulations of OVA (free OVA, F8-S/O, and F12-S/O) for 24 h. Values are the mean ± SD (*n* = 3).

To assess the safety profile of the newly developed S/O nanodispersions, in vitro cytotoxicity was evaluated using the CCK-8 assay in HeLa cells (Figure 5). Cells were treated with various concentrations (12.5, 25, and 50 μg/mL) of free OVA or S/O nanodispersions (F8-S/O and F12-S/O). Across all tested concentrations, the mean cell viability remained above 90%, suggesting negligible cytotoxicity and good biocompatibility of the formulations. These results indicate that newly formulated S/O nanodispersions are biocompatible and suitable for transdermal applications. HeLa cells were employed as a standardized in vitro model for the preliminary assessment of the cytotoxicity of the formulations. The primary objective of this assay was to establish the baseline of cell viability prior to in vivo evaluation. We acknowledge that normal skin-derived cells such as human keratinocytes or primary fibroblasts would offer a more physiologically relevant model for transdermal formulations. These specialized models will be incorporated in our future work.

### 2.4. In Vivo Skin Irritation Evaluation

**Figure 6:**
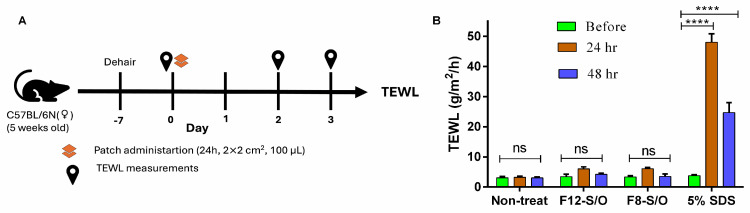
(**A**) In vitro skin irritation study design. (**B**) TEWL values measured before application, and 24 h and 48 h after patch removal. Statistical significance was assessed using Tukey’s multiple comparison test: **** *p* < 0.0001. Values are the mean ± SD (*n* = 3). ns = non-significant.

Trans-epidermal water loss (TEWL) was determined to evaluate the skin irritation potential of the S/O nanodispersions. Initial TEWL values were recorded for all mouse groups before the application of the formulations. At 24 h post-application, the patches were removed, and TEWL values were re-measured to assess the change in water loss. Although a slight increase in TEWL was observed, the differences were not statistically significant (Figure 6). Subsequent measurements at 48 h showed that TEWL values had recovered to their initial levels, indicating recovery of the skin’s barrier functions. However, significant TEWL values were observed after 48 h, when the mouse groups were treated with 5% SDS in PBS. These findings indicate that S/O nanodispersions caused the minimal and reversible disruption of skin integrity, supporting their safe use for transdermal applications.

### 2.5. In Vivo Immunization and OVA-Specific Antibody Responses

**Figure 7:**
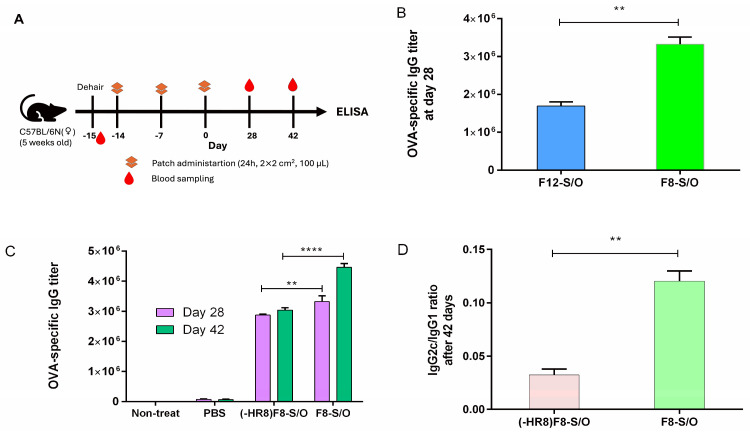
(**A**) Experimental design for the in vivo immunization of C57BL/6N mice. (**B**) Results of ELISA measurements and comparisons between S/O nanodispersions (F8-S/O and F12-S/O). (**C**) OVA-specific IgG responses at 28 days and 42 days after the third immunization. (**D**) OVA-specific IgG1 and IgG2c responses after the third immunization. Statistical significance is assessed using a paired *t*-test (**B**,**D**) and Tukey’s multiple comparison test (**C**): ** *p* < 0.01 and **** *p* < 0.0001. Values are the mean ± SD (*n* = 3).

The immunization efficacy of the developed S/O formulations was evaluated by administering vaccine patches transdermally to C57BL/6N mice three consecutive times according to a defined schedule (Figure 7A). After the third immunization, blood samples were collected at 28 days and 42 days for antibody analysis. Immune responses induced by S/O nanodispersions were determined by measuring serum anti-OVA antibody levels by an enzyme-linked immunosorbent assay (ELISA). Initial observations at 28 days after the third immunization indicated that F8-S/O nanodispersions induced approximately 2-fold higher OVA-specific IgG titers compared with F12-S/O nanodispersions. This enhanced antibody production observed with F8-S/O nanodispersions suggested improved antigen presentation (Figure 7B). This observation aligns with the findings of Kitaoka et al. who demonstrated that increasing the amount of surfactant reduced antigen presentation and antibody responses [40]. At 28 and 42 days after the third immunization, F8-S/O nanodispersions exhibited approximately 46-fold and 68-fold higher OVA-specific IgG titers, respectively, compared with their mixture in PBS, indicating the successful induction of antigen-specific immune responses via the transdermal route (Figure 7C). These results confirm the ability of the S/O nanodispersions to effectively deliver OVA across the skin barrier and induce robust immune responses via the transdermal route. To elucidate the role of the DC-targeting HR8 peptide, an S/O nanodispersion without HR8 peptide was used as a control. Enhancement of immune responses was observed between the F8-S/O nanodispersion and (−HR8) F8-S/O nanodispersion, with the F8-S/O nanodispersion showing approximately 1.2-fold and 1.5-fold higher IgG titers at 28 and 42 days, respectively (Figure 7C). The enhanced immune response exhibited by the F8-S/O nanodispersion may be associated with the inclusion of the HR8 peptide which was previously reported by Zheng et al. as a DC-targeting peptide [15]. The authors demonstrated the DC-targeting ability of the HR8 peptide and showed that it could enhance the antigen-specific immune responses. However, the exact mechanism of HR8-mediated DC-targeting was not directly addressed in the present study. Therefore, the proposed role of HR8 peptide in facilitating DC-targeting is based on previous studies and should be regarded as a plausible explanation to be evaluated in future investigations using cellular uptake, receptor-binding and fluorescence colocalization assays. To further characterize the type of immunological responses, IgG subclasses were investigated, including IgG1 and IgG2c. IgG1 is generally associated with Th2-mediated humoral immunity, whereas IgG2c represents Th1-driven cellular immunological responses. The ratio of IgG2c/IgG1 can be used to determine Th1 or Th2-type immune responses [34]. In this study, OVA-specific IgG1 levels were higher than IgG2c levels in the sera of immunized mice, indicating a predominantly Th2-biased immune response. However, the inclusion of HR8 peptide into the S/O nanodispersion led to an approximately 2.5-fold increase in IgG2c levels, suggesting a shift toward a more Th1-biased immune response compared with S/O nanodispersion without HR8 peptide (Figure S1). Consistently, for F8-S/O nanodispersion, when the IgG2c/IgG1 ratio increased, the Th1/Th2 balance shifted in favor of Th1-driven immune responses, indicating enhanced cellular immunity (Figure 7D). This shift toward the Th1 immunity is particularly important for antitumor immunity because Th1 responses are associated with the activation of cytotoxic T lymphocytes, which improve tumor clearance [19]. Previous studies have demonstrated that DC-targeting immunization strategies enhanced cellular immune responses and produced sustained antitumor effects in animal models [45,46,47]. In line with these findings, Zheng et al. reported that a DC-targeting HR8 peptide significantly enhanced cellular immune responses and anti-tumor activity following its subcutaneous administration [15]. However, the present study demonstrated that the transdermal co-delivery of HR8 peptide and a model antigen OVA using S/O nanodispersion promoted a shift toward a more Th1-biased immune response, which supports the effectiveness of this simple and patient-compliant, needle-free approach. Although further studies are required to fully elucidate the mechanistic role of the HR8 peptide in modulating immune responses, including its interactions with dermal DCs and intracellular trafficking pathways, the present results clearly demonstrate that the S/O nanodispersion approach is capable of inducing robust and functionally relevant immune responses.

## 3. Materials and Methods

### 3.1. Materials

OVA was obtained from Sigma-Aldrich (St. Louis, MO, USA) and HR8 peptide (sequence: HLHKHHLR; molecular weight: 1077.27 Da) was purchased from GenScript (Piscataway, NJ, USA) with a purity of 96.4%. Isopropyl myristate (IPM) and cyclohexane were acquired from TCI (Tokyo, Japan) and Wako (Kyoto, Japan), respectively. Sucrose erucate (ER-290) and sucrose laurate (L-195) were kindly provided by Mitsubishi Chemical Foods (Tokyo, Japan). A Bicinchoninic Acid (BCA) Protein Assay Kit was purchased from Thermo Fisher Scientific (Waltham, MA, USA). CpG ODN 1826: 5′-tccatga**cg**ttcctga**cg**tt-3′ (20 mer) was acquired from Invivogen (Carlsbad, CA, USA). Horseradish peroxidase (HRP)-conjugated rabbit anti-mouse IgG, IgG1, and IgG2c antibodies were sourced from Rockland Immuno-Chemicals (Gilbertsville, PA, USA).

### 3.2. Cells

HeLa cells were sourced from the RIKEN cell bank (Tsukuba, Japan). Fetal bovine serum (FBS), Dulbecco’s Modified Eagle Medium (DMEM), and antibiotic–antimycotic were obtained from Thermo Fisher Scientific (Waltham, MA, USA). Dulbecco’s phosphate-buffered saline (DPBS) and trypsin/ethylenediaminetetraacetic acid solution were acquired from Nacalai Tesque (Kyoto, Japan). A CCK-8 assay kit was acquired from Dojindo Molecular Technologies, Inc. (Kumamoto, Japan).

### 3.3. Animals

For immunization studies and skin irritation test, 5-week-old C57BL/6N female mice were obtained from Kyudo Corporation (Saga, Japan) and raised under standard conditions.

### 3.4. S/O Nanodispersions and Characterization

S/O nanodispersions were formulated following a previously reported protocol with minor modifications [18,48]. Briefly, OVA and HR8 peptides were mixed at molar ratios of 1:10, 1:20, 1:30, and 1:40 in 2 mL of Milli-Q water and the zeta potential was determined using a laser Doppler electrophoresis method with a Zetasizer NanoZS (Malvern, Worcestershire, UK). Then, 200 µL of CpG ODN 1826 (0.05 mg/mL) was added to 2 mL of the OVA-HR8 solution. Next, 2.2 mL of this solution was added to 4.4 mL of cyclohexane containing sucrose erucate (ER-290) in a glass vial (1:2, *v*/*v*). The resulting mixture was then homogenized at 26,000 rpm for 2 min using a Polytron homogenizer PT3100D (Kinematica AG, Lucerne, Switzerland) to form water-in-oil (W/O) emulsions. The prepared emulsions were immediately frozen in liquid nitrogen for 20 min, followed by lyophilization for 18 h using a freeze dryer (FD5N; EYELA, Tokyo, Japan) to yield a protein–surfactant complex. Subsequently, 1 mL of IPM was added to the obtained solid complex and the mixture was vortexed at room temperature and stirred at 500 rpm for 12 h to form the final S/O nanodispersion. The complexation between the OVA-HR8 complex and the surfactant was not evaluated directly, but the hydrodynamic diameter of the particles, PDI and the stability results indirectly supported the successful formation of stable S/O nanodispersions. As a comparison study, S/O nanodispersions were also prepared using sucrose laurate (L-195) under identical conditions (Table S2). The OVA concentration in IPM was fixed at 1 mg/mL. The hydrodynamic diameter of particles (Table S3) of the resulting nanodispersions was analyzed by dynamic light scattering (DLS) with a Zetasizer NanoZS (Malvern, Worcestershire, UK).

For in vitro fluorescence microscopic analysis of S/O nanodispersions in mouse skin, formulations were prepared using FITC-labeled OVA (1.0 mg/mL) combined with HR8 peptide and a surfactant (Table S5).

### 3.5. Cell Viability Assay

Cell viability was evaluated using HeLa cells following a previously reported protocol with slight modifications [49]. Cells were cultured to approximately 70–80% confluence and harvested by trypsinization. Subsequently, the cells were plated into a 96-well flat-bottomed plate at 5 × 10^4^ cells per well (100 μL) and cultured in D-MEM containing 10% fetal bovine serum and 1% antibiotic-antimycotic at 37 °C in a CO_2_ incubator for 24 h. The medium was then exchanged for fresh medium containing various concentrations of free OVA or S/O nanodispersions (F8-S/O and F12-S/O) at OVA concentrations of 12.5, 25, and 50 μg/mL, followed by incubation for an additional 24 h. Cell viability was then assessed using a CCK-8 assay in accordance with the manufacturer’s protocol. Untreated cells were used as the control and all experiments were conducted in triplicate. Cell viability was calculated according to the following equation:

Cell viability [%] = [(As − Ab)/(Ac − Ab)] × 100

where As represents the absorbance of the sample, Ac the absorbance of the negative control, and Ab the absorbance of the blank.

### 3.6. In Vitro Protein (OVA) Release Study

The release of OVA from S/O nanodispersions into phosphate-buffered saline (PBS) was evaluated following a previously reported method [40]. Briefly, 150 μL of the S/O nanodispersion was added to 600 μL PBS and stirred at 37 °C for 24 h. At predetermined time points (1, 3, 12, and 24 h), aliquots (20 μL) were collected, and the released OVA concentration was quantified using a BCA assay with a kit purchased from Thermo Fisher Scientific (Waltham, MA, USA). All procedures were conducted in accordance with the manufacturer’s instructions and following a previously reported method [18,34,40].

### 3.7. In Vitro Skin Permeation Study

For the fluorescence microscopy-based assessment of transdermal delivery, OVA was labeled with fluorescein isothiocyanate (FITC) according to a previously reported method [50]. The in vitro skin permeation of FITC-OVA-loaded S/O formulations (1 mg/mL) was assessed using excised mouse skin mounted on a Franz diffusion cell (FDC), according to a previously reported method. Briefly, frozen (−80 °C) mouse skin was thawed at room temperature, and cut to an appropriate size (2 cm × 2 cm) and immediately mounted on an FDC (diffusion area: 0.785 cm^2^), with the stratum corneum facing the donor compartment and the dermis facing the receptor compartment. Initially, the receptor chamber was filled with 4 mL of PBS (pH 7.4) and equipped with a magnetic stirrer. To remove air bubbles, 1 mL of PBS was administered through the branch tube into the FDC. The donor compartment was filled with 200 μL of S/O nanodispersion or PBS solution consisting of a mixture of FITC-OVA, CpG ODN 1826 and HR8 peptide as control. This study was conducted at 32.5 °C for 6 h under constant stirring (500 rpm). To evaluate the topical delivery, the treated skins were removed from the FDC, thoroughly washed repeatedly with 20% ethanol and sectioned into small pieces using a scalpel. The skin pieces were then immersed in 1 mL of extraction solution comprising PBS, acetonitrile, and methanol at a ratio of 2:1:1 (*v*/*v*/*v*) and the extraction was conducted at room temperature for 18 h under continuous shaking. The transdermal (across-skin) delivery of FITC-OVA in the receiver phase as well as the topical (into-skin) delivery of FITC-OVA from the extracted skin samples was quantified by determining the fluorescence intensity at 485–535 nm using a Varioskan LUX multimode microplate reader (Thermo Fisher Scientific).

### 3.8. In Vivo Skin Irritation Study

In vivo skin irritation induced by the S/O formulations was evaluated by determining the trans-epidermal water loss (TEWL), expressed in g/m^2^·h. C57BL/6N mice were anesthetized for 10 min prior to the experiment, and the TEWL value of the dehaired mice was recorded using a VAPOSCAN (Asch Japan Co., Ltd., Tokyo, Japan) (*n* = 3). Following the application and subsequent removal of the formulation-loaded patch, TEWL measurements were taken at 24 and 48 h. For comparison, the TEWL measurements were also performed in mice treated with 5% sodium dodecyl sulfate (SDS) in PBS as a positive control and untreated mice as a negative control at the same time points.

### 3.9. In Vivo Immunization and Measurements of IgG-Specific Antibodies

An isoflurane vaporizer chamber was used to anesthetize C57BL/6N mice for adequate sedation and dehaired before immunization. Ovalbumin (100 µg per mouse) was administered by topical transdermal application to the dorsal skin of mice three times at one-week intervals. Homemade patches consisting of two layers of gauze (approximately 2 × 2 cm^2^, Hakujuji Co., Ltd., Tokyo, Japan) were used as the application matrix for all formulations. Each patch was loaded with 100 µL of the test S/O nanodispersions (F8-S/O and F12-S/O) or the corresponding control formulation before being applied to the dorsal skin. The control formulations included a PBS solution containing OVA, HR8 peptide, and adjuvant as well as an S/O nanodispersion without the HR8 peptide. The patches were removed after 24 h and the same procedures were followed for the second and third immunizations. Blood samples were collected from the tail vein of individual mice 1 day before the first immunization and on days 28 days and 42 after the third immunization for subsequent immunological analyses. For this purpose, the sera were separated by centrifugation at 3500 rpm for 10 min and anti-OVA IgG, IgG1, and IgG2c were evaluated using ELISA as previously reported [50].

### 3.10. Statistical Analysis

GraphPad Prism software (Version 6.05) was used for statistical analysis. Statistical significances were evaluated by one-way analysis of variance (ANOVA) followed by Tukey’s multiple comparison test and paired *t*-test. Statistical significance is indicated as * *p* < 0.05, ** *p* < 0.01, *** *p* < 0.001, and **** *p* < 0.0001. Data are presented as the mean ± standard deviation of the mean.

## 4. Conclusions

This study demonstrated that the incorporation of a DC targeting HR8 peptide into an S/O nanodispersion provided an effective platform for transdermal immunization. The newly developed formulation successfully delivered OVA and induced significantly higher OVA-specific IgG responses compared with PBS solution, highlighting improved antigen delivery. S/O nanodispersions containing the HR8 peptide exhibited approximately 1.5-fold higher OVA-specific IgG responses than those without HR8 peptides. Importantly, S/O nanodispersion containing the HR8 peptide promoted an approximately 2.5-fold increase in IgG2c levels, indicating a shift toward a more Th1-biased immune response. Further investigation of the HR8 peptide-mediated DC-targeting strategies may provide deeper insights into its immunological advantages. Additionally, extending this platform to other antigens might broaden its applicability as a versatile transdermal immunization strategy. Future studies focusing on the therapeutic evaluation of this formulation will help to fully realize the clinical potential of this approach. Overall, these findings highlight the potential of S/O nanodispersions as a needle-free vaccine delivery platform capable of eliciting robust immune responses.

## Data Availability

The data curated or analyzed for this study are presented in the article and supporting data have been attached as the Supplementary Materials.

## Supplementary Materials

The following supporting information can be downloaded at https://www.mdpi.com/article/10.3390/molecules31152668/s1, Figure S1: OVA-specific IgG2c responses; Table S1: Zeta potentials measurement; Table S2: Compositions of F1~F12 solid-in-oil (S/O) nanodispersions; Table S3: Hydrodynamic diameter of particles and polydispersity index (PDI) of F1~F12 solid-in-oil (S/O) nanodispersions; Table S4: Hydrodynamic diameter of particles and PDI of F8-S/O and F12 S/O nanodispersions until 90 days; Table S5: composition of S/O nanodispersions loaded with FITC-OVA.

## References

[1] Combadiere B., Liard C. (2011). Transcutaneous and Intradermal Vaccination. Hum. Vaccines.

[2] Azmal M., Miah M.M., Prima F.S., Paul J.K., Haque A.S.N.B., Ghosh A. (2025). Advances and Challenges in Cancer Immunotherapy: Strategies for Personalized Treatment. Semin. Oncol..

[3] Ita K. (2016). Transdermal Delivery of Vaccines—Recent Progress and Critical Issues. Biomed. Pharmacother..

[4] Illum L. (2002). Nasal Drug Delivery: New Developments and Strategies. Drug Discov. Today.

[5] Jabbal-Gill I. (2010). Nasal Vaccine Innovation. J. Drug Target..

[6] Mitragotri S. (2005). Immunization without Needles. Nat. Rev. Immunol..

[7] Seid R.C., Look J.L., Ruiz C., Frolov V., Flyer D., Schafer J., Ellingsworth L. (2012). Transcutaneous Immunization with Intercell’s Vaccine Delivery System. Vaccine.

[8] Svenskaya Y.I., Genina E.A., Parakhonskiy B.V., Lengert E.V., Talnikova E.E., Terentyuk G.S., Utz S.R., Gorin D.A., Tuchin V.V., Sukhorukov G.B. (2019). A Simple Non-Invasive Approach toward Efficient Transdermal Drug Delivery Based on Biodegradable Particulate System. ACS Appl. Mater. Interfaces.

[9] Zhao T., Cai Y., Jiang Y., He X., Wei Y., Yu Y., Tian X. (2023). Vaccine Adjuvants: Mechanisms and Platforms. Signal Transduct. Target. Ther..

[10] Alexander A., Dwivedi S., Ajazuddin, Giri T.K., Saraf S., Saraf S., Tripathi D.K. (2012). Approaches for Breaking the Barriers of Drug Permeation through Transdermal Drug Delivery. J. Control. Release.

[11] Joffre O.P., Segura E., Savina A., Amigorena S. (2012). Cross-Presentation by Dendritic Cells. Nat. Rev. Immunol..

[12] Bal S.M., Ding Z., Van Riet E., Jiskoot W., Bouwstra J.A. (2010). Advances in Transcutaneous Vaccine Delivery: Do All Ways Lead to Rome?. J. Control. Release.

[13] Kvedaraite E., Ginhoux F. (2022). Human Dendritic Cells in Cancer. Sci. Immunol..

[14] Li N., Peng L.H., Chen X., Nakagawa S., Gao J.Q. (2011). Transcutaneous Vaccines: Novel Advances in Technology and Delivery for Overcoming the Barriers. Vaccine.

[15] Zheng J., Wang M., Pang L., Wang S., Kong Y., Zhu X., Zhou X., Wang X., Chen C., Ning H. (2024). Identification of a Novel DEC-205 Binding Peptide to Develop Dendritic Cell-Targeting Nanovaccine for Cancer Immunotherapy. J. Control. Release.

[16] Gou S., Liu W., Wang S., Chen G., Chen Z., Qiu L., Zhou X., Wu Y., Qi Y., Gao Y. (2021). Engineered Nanovaccine Targeting Clec9a+ Dendritic Cells Remarkably Enhances the Cancer Immunotherapy Effects of STING Agonist. Nano Lett..

[17] Schreibelt G., Bol K.F., Westdorp H., Wimmers F., Aarntzen E.H.J.G., Duiveman-De Boer T., Van De Rakt M.W.M.M., Scharenborg N.M., De Boer A.J., Pots J.M. (2016). Effective Clinical Responses in Metastatic Melanoma Patients after Vaccination with Primary Myeloid Dendritic Cells. Clin. Cancer Res..

[18] Kitaoka M., Imamura K., Hirakawa Y., Tahara Y., Kamiya N., Goto M. (2013). Needle-Free Immunization Using a Solid-in-Oil Nanodispersion Enhanced by a Skin-Permeable Oligoarginine Peptide. Int. J. Pharm..

[19] Luo W., Hu J., Xu W., Dong J. (2022). Distinct Spatial and Temporal Roles for Th1, Th2, and Th17 Cells in Asthma. Front. Immunol..

[20] Sosa Cuevas E., Valladeau-Guilemond J., Mouret S., Roubinet B., de Fraipont F., Landemarre L., Charles J., Bendriss-Vermare N., Chaperot L., Aspord C. (2022). Unique CLR Expression Patterns on Circulating and Tumor-Infiltrating DC Subsets Correlated with Clinical Outcome in Melanoma Patients. Front. Immunol..

[21] Wang F., Ullah A., Fan X., Xu Z., Zong R., Wang X., Chen G. (2021). Delivery of Nanoparticle Antigens to Antigen-Presenting Cells: From Extracellular Specific Targeting to Intracellular Responsive Presentation. J. Control. Release.

[22] Lewis J.S., Zaveri T.D., Crooks C.P., Keselowsky B.G. (2012). Microparticle Surface Modifications Targeting Dendritic Cells for Non-Activating Applications. Biomaterials.

[23] Cruz L.J., Rosalia R.A., Kleinovink J.W., Rueda F., Löwik C.W.G.M., Ossendorp F. (2014). Targeting Nanoparticles to CD40, DEC-205 or CD11c Molecules on Dendritic Cells for Efficient CD8+ T Cell Response: A Comparative Study. J. Control. Release.

[24] Li M., Sharma F.H., Chen Y.L., Araneda M.E., Hammett A., Miller D., Pearce L., Chen K.H.E. (2025). Development of a Novel Assay for Antigen Presentation Measurement. Sci. Rep..

[25] Zhu B.T. (2025). Molecular Mechanism for the Selective Presentation of Antigenic Peptides by Major Histocompatibility Complex Class I and Class II Molecules: A Hypothesis. Curr. Issues Mol. Biol..

[26] Baljon J.J., Wilson J.T. (2022). Bioinspired Vaccines to Enhance MHC Class-I Antigen Cross-Presentation. Curr. Opin. Immunol..

[27] Wang R.F., Wang H.Y. (2002). Enhancement of Antitumor Immunity by Prolonging Antigen Presentation on Dendritic Cells. Nat. Biotechnol..

[28] Mitsui H., Inozume T., Kitamura R., Shibagaki N., Shimada S. (2006). Polyarginine-Mediated Protein Delivery to Dendritic Cells Presents Antigen More Efficiently onto MHC Class I and Class II and Elicits Superior Antitumor Immunity. J. Investig. Dermatol..

[29] Bolhassani A. (2019). Improvements in Chemical Carriers of Proteins and Peptides. Cell Biol. Int..

[30] Kayraklioglu N., Horuluoglu B., Klinman D.M. (2021). CpG Oligonucleotides as Vaccine Adjuvants. Methods Mol. Biol..

[31] Scheiermann J., Klinman D.M. (2014). Clinical Evaluation of CpG Oligonucleotides as Adjuvants for Vaccines Targeting Infectious Diseases and Cancer. Vaccine.

[32] Kastenmüller W., Kastenmüller K., Kurts C., Seder R.A. (2014). Dendritic Cell-Targeted Vaccines—Hope or Hype?. Nat. Rev. Immunol..

[33] Darvishha S., Amiri S. (2019). (Trans) Dermal Insulin Delivery Based on Polymeric Systems. Int. J. Polym. Mater. Polym. Biomater..

[34] Kitaoka M., Naritomi A., Hirakawa Y., Kamiya N., Goto M. (2015). Transdermal Immunization Using Solid-in-Oil Nanodispersion with CpG Oligodeoxynucleotide Adjuvants. Pharm. Res..

[35] Ko J., Kim J.U., Choi S., Kim Y.S., Park S.B., Kim J.Y., Kim H.J., Lee Y.S., An Y.H., Hwang N.S. (2024). Recent Advances in Nanotechnology-Mediated Noninvasive Transdermal and Topical Delivery of Proteins. Small Sci..

[36] Sadaf A., Sinha R., Ekka M.K. (2022). Ionic Liquid-Mediated Skin Technologies: Recent Advances and Prospects. Curr. Res. Biotechnol..

[37] Luo Z., Li P., Deng J., Gao N., Zhang Y., Pan H., Liu L., Wang C., Cai L., Ma Y. (2013). Cationic Polypeptide Micelle-Based Antigen Delivery System: A Simple and Robust Adjuvant to Improve Vaccine Efficacy. J. Control. Release.

[38] Toyofuku K., Wakabayashi R., Kawaguchi Y., Kamiya N., Goto M. (2025). Biocompatible Ionic Liquid-Based Nanoparticles for Effective Skin Penetration and Intracellular Uptake of Antisense Oligonucleotides. RSC Pharm..

[39] Tanaka K., Masaki H., Minamihata K., Wakabayashi R., Kawaguchi Y., Kamiya N., Goto M. (2025). Needle-Free Transdermal Patch for Influenza Vaccination. ACS Appl. Bio Mater..

[40] Kitaoka M., Imamura K., Hirakawa Y., Tahara Y., Kamiya N., Goto M. (2013). Sucrose Laurate-Enhanced Transcutaneous Immunization with a Solid-in-Oil Nanodispersion. MedChemComm.

[41] Adams J.R., Haughney S.L., Mallapragada S.K. (2015). Effective Polymer Adjuvants for Sustained Delivery of Protein Subunit Vaccines. Acta Biomater..

[42] Hansen S., Lehr C.M. (2012). Nanoparticles for Transcutaneous Vaccination. Microb. Biotechnol..

[43] Mansour R.S.H., Sallam A.A., Hamdan I.I., Khalil E.A., Yousef I. (2017). Elucidation of Penetration Enhancement Mechanism of Emu Oil Using FTIR Microspectroscopy at EMIRA Laboratory of SESAME Synchrotron. Spectrochim. Acta A Mol. Biomol. Spectrosc..

[44] Tahara Y., Honda S., Kamiya N., Piao H., Hirata A., Hayakawa E., Fujii T., Goto M. (2008). A Solid-in-Oil Nanodispersion for Transcutaneous Protein Delivery. J. Control. Release.

[45] Tang X.D., Lü K.L., Yu J., Du H.J., Fan C.Q., Chen L. (2022). In Vitro and In Vivo Evaluation of DC-Targeting PLGA Nanoparticles Encapsulating Heparanase CD4+ and CD8+ T-Cell Epitopes for Cancer Immunotherapy. Cancer Immunol. Immunother..

[46] Wu D., Liu Z., Feng Y., Tang F., Li S., Zhang X., Li H., Liu Q., Zhang L., Liu Q. (2023). Development and Characterization of DEC-205 Receptor Targeted Potentilla Anserina L Polysaccharide PLGA Nanoparticles as an Antigen Delivery System to Enhance In Vitro and In Vivo Immune Responses in Mice. Int. J. Biol. Macromol..

[47] Feng Y., Fan J., Wu D., Liu Q., Li H., Zhang X., Li S., Tang F., Liu Z., Zhang L. (2023). DEC-205 Receptor Targeted Poly(Lactic-Co-Glycolic Acid) Nanoparticles Containing Eucommia Ulmoides Polysaccharide Enhances the Immune Response of Foot-and-Mouth Disease Vaccine in Mice. Int. J. Biol. Macromol..

[48] Zhang Y., Pan W., Wang D., Wang H., Hou Y., Zou M., Piao H. (2023). Solid-in-oil nanodispersion as a novel topical transdermal delivery to enhance stability and skin permeation and retention of hydrophilic drugs l-ascorbic acid. Eur. J. Pharm. Biopharm..

[49] Li S., Yang K., Cao W., Guo R., Liu Z., Zhang J., Fan A., Huang Y., Ma C., Li L. (2023). Tanshinone IIA enhances the therapeutic efficacy of mesenchymal stem cells derived exosomes in myocardial ischemia/reperfusion injury via up-regulating miR-223-5p. J. Control. Release.

[50] Chowdhury M.R., Moshikur R.M., Wakabayashi R., Moniruzzaman M., Goto M. (2021). Biocompatible Ionic Liquids Assisted Transdermal Co-Delivery of Antigenic Protein and Adjuvant for Cancer Immunotherapy. Int. J. Pharm..

