# Supplementary material for: Enhanced Transdermal Immunization via Solid-in-Oil Nanodispersions Incorporating Dendritic Cell-Targeting Peptide
Source: Molecules. 2026 Jul 31;31(15):2668. doi: 10.3390/molecules31152668 (PMC13468327; doi:10.3390/molecules31152668)
Supplement: Supplementary file 1 [file molecules-31-02668-s001.zip › molecules-4460650-supplementary.pdf]

Supplementary materials

Enhanced Transdermal Immunization via Solid-in-Oil Nanodispersions Incorporating Dendritic Cell-Targeting Peptide

Md. Samiul Islam[1,2], Md. Shahin Sarker[1,3], Yoshirou Kawaguchi[1], Rie Wakabayashi[1], Noriho Kamiya[1,4], Muhammad Moniruzzaman[5], and Masahiro Goto[1,4*]

[1] Department of Applied Chemistry, Graduate School of Engineering, Kyushu University, 744 Motooka, Fukuoka 819-0395, Japan

[2] Department of Pharmaceutical Technology, Faculty of Pharmacy, University of Dhaka, Dhaka-1000, Bangladesh

[3] Department of Pharmacy, Jashore University of Science and Technology, Jashore, Bangladesh

[4] Center for Future Chemistry, Kyushu University, Motooka 744, Nishi-ku, Fukuoka 819-0395, Japan.

[5] Mechanical Engineering Technology, CUNY New York City College of Technology, 186 Jay Street, Brooklyn, NY-11201, USA

[*]Corresponding author:

Masahiro Goto

Address: Department of Applied Chemistry, Graduate School of Engineering, Kyushu University, 744 Motooka, Fukuoka 819-0395, Japan

Tel: +81 92 802 2806; Fax: +81 92 802 2810

E-mail: m-goto@mail.cstm.kyushu-u.ac.jp

TABLE OF CONTENTS

1.	Zeta potentials measurement	Table S1
2.	Compositions of F1~F12 solid-in-oil (S/O) nanodispersions	Table S2
3.	Hydrodynamic diameter of particles and polydispersity index (PDI) of F1~F12 solid-in-oil (S/O) nanodispersions	Table S3
4.	Hydrodynamic diameter of particles and PDI of F8-S/O and F12 S/O nanodispersions until 90 days	Table S4
5.	OVA-specific IgG2c responses	Figure S1
6.	Composition of S/O nanodispersions loaded with FITC-OVA	Table S5

1. Zeta potentials measurement

Table S1. Zeta potentials measurement of the mixture of OVA and HR8 peptide in Milli-Q water

OVA and HR8 (molar ratio)	Zeta (ζ) potential (mV) ± SD at room temperature
Only OVA	-28.0 ± 6.3
Only HR8	24.4 ± 5.7
OVA:HR8 = 1:10	0.2 ± 0.1
OVA:HR8 = 1:20	9.1 ± 0.8
OVA:HR8 = 1:30	15.3 ± 2.3
OVA:HR8 = 1:40	18.2 ± 2.9

2. S/O nanodispersions and characterization

Prior to the preparation of S/O nanodispersions 1 mg OVA was added to 0.25, 0.50, 0.75 & 1 mg HR8 peptide. The molar ratio of OVA and HR8 peptide was 1:10, 1:20, 1:30 & 1:40 respectively.

Table S2. Compositions of F1~F12-S/O nanodispersions

S/O nano-dispersion	HR-8 peptide (mg)	OVA (mg)	CpG-ODN 1826 (µg)	L-195 (mg)	ER-290 (mg)
F1	0.25	1.00	10	50	-
F2	0.50	1.00	10	50	-
F3	0.75	1.00	10	50	-
F4	1.00	1.00	10	50	-
F5	0.25	1.00	10	-	50
F6	0.50	1.00	10	-	50
F7	0.75	1.00	10	-	50
F8	1.00	1.00	10	-	50
F9	0.25	1.00	10	-	100
F10	0.50	1.00	10	-	100
F11	0.75	1.00	10	-	100
F12	1.00	1.00	10	-	100

Table S3. Hydrodynamic diameter of particles and polydispersity index (PDI) of F1~F12 S/O nanodispersions

S/O nanodispersions	**Day 1**	
	Z-average (nm)	PDI
F1	510.7 ± 24.06	0.159 ± 0.059
F2	614.3 ± 72.17	0.247 ± 0.175
F3	698.6 ± 62.36	0.223 ± 0.071
F4	1449 ± 247.2	0.506 ± 0.451
F5	452.2 ± 41.21	0.988 ± 0.026
F6	263.2 ± 08.70	0.614 ± 0.111
F7	266.8 ± 37.05	0.791 ± 0.111
F8	199.0 ± 10.90	0.097 ± 0.082
F9	204.5 ± 22.24	0.766 ± 0.003
F10	194.6 ± 13.27	0.589 ± 0.039
F11	260.9 ± 37.17	0.526 ± 0.084
F12	193.2 ± 33.12	0.343 ± 0.066

Table S4. Hydrodynamic diameter of particles and PDI of F8-S/O and F12 S/O nanodispersions until 90 days

Dispersion	**Day 1**		**Day 7**		**Day 15**		**Day 30**		**Day 60**		**Day 90**	
	Z-avg (nm)	PDI	Z-avg (nm)	PDI	Z-avg (nm)	PDI	Z-avg (nm)	PDI	Z-avg (nm)	PDI	Z-avg (nm)	PDI
**F8-S/O**	199.0 ± 10.90	0.097 ± 0.082	206.2 ± 4.581	0.078 ± 0.057	209.9 ± 3.579	0.112 ± 0.048	208.6 ± 2.755	0.165 ± 0.045	191.2 ± 9.409	0.166 ± 0.053	176.1 ± 9.50	0.229 ± 0.020
**F12-S/O**	193.2 ± 33.12	0.343 ± 0.066	200.1 ± 13.82	0.260 ± 0.094	211.0 ± 9.643	0.319 ± 0.041	177.6 ± 9.153	0.345 ± 0.061	149.2 ± 6.483	0.163 ± 0.088	165.6 ± 10.15	0.248 ± 0.028

3. *In vivo* immunization and OVA-specific IgG2c responses

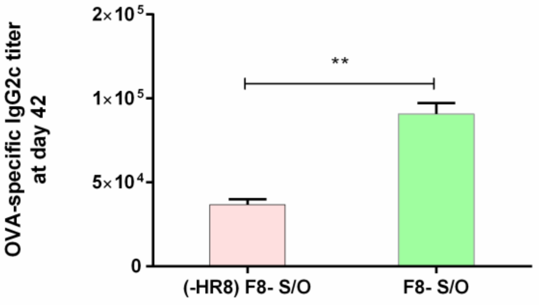

Figure S1: OVA-specific IgG2c responses for 42 days after third immunization. Statistical significance was assessed using paired t test $*p < 0.05$, $**p < 0.01$, $***p < 0.001$ and $****p < 0.0001$, mean ± SD (N = 3).

4. S/O nanodispersions loaded with FITC-OVA

Table S5. Composition of S/O loaded with FITC-OVA

S/O nano dispersion	HR-8 peptide (mg)	FITC-OVA (mg)	CpG-ODN 1826 (μg)	ER-290 (mg)
F8 with FITC-OVA	1.00	1.00	10	50
F12 with FITC-OVA	1.00	1.00	10	100
